# A Web-Based Cognitive Behavior Therapy Intervention to Improve Social and Occupational Functioning in Adults With Type 2 Diabetes (The SpringboarD Trial): Randomized Controlled Trial

**DOI:** 10.2196/12246

**Published:** 2019-05-21

**Authors:** Janine Clarke, Samineh Sanatkar, Peter Andrew Baldwin, Susan Fletcher, Jane Gunn, Kay Wilhelm, Lesley Campbell, Nicholas Zwar, Mark Harris, Helen Lapsley, Dusan Hadzi-Pavlovic, Helen Christensen, Judy Proudfoot

**Affiliations:** 1 Black Dog Institute Sydney Australia; 2 School of Psychiatry UNSW Sydney Sydney Australia; 3 The Black Dog Institute Sydney Australia; 4 Department of General Practice University of Melbourne Melbourne Australia; 5 Diabetes and Metabolism Division Garvan Institute of Medical Research Sydney Australia; 6 School of Medicine University of Wollongong Wollongong Australia; 7 Centre for Primary Health Care and Equity UNSW Sydney Sydney Australia

**Keywords:** type 2 diabetes, depression, internet

## Abstract

**Background:**

Depressive symptoms are common in people with type 2 diabetes mellitus (T2DM). Effective depression treatments exist; however, access to psychological support is characteristically low. Web-based cognitive behavioral therapy (CBT) is accessible, nonstigmatizing, and may help address substantial personal and public health impact of comorbid T2DM and depression.

**Objective:**

The aim of this study was to evaluate the Web-based CBT program, myCompass, for improving social and occupational functioning in adults with T2DM and mild-to-moderate depressive symptoms. myCompass is a fully automated, self-guided public health treatment program for common mental health problems. The impact of treatment on depressive symptoms, diabetes-related distress, anxiety symptoms, and self-care behavior was also examined.

**Methods:**

Participants with T2DM and mild-to-moderate depressive symptoms (N=780) were recruited online via Google and Facebook advertisements targeting adults with T2DM and via community and general practice settings. Screening, consent, and self-report scales were all self-administered online. Participants were randomized using double-blind computerized block randomization to either myCompass (n=391) for 8 weeks plus a 4-week tailing-off period or an active placebo intervention (n=379). At baseline and postintervention (3 months), participants completed the Work and Social Adjustment Scale, the primary outcome measure. Secondary outcome measures included the Patient Health Questionnaire-9 item, Diabetes Distress Scale, Generalized Anxiety Disorder Questionnaire-7 item, and items from the Self-Management Profile for Type 2 Diabetes.

**Results:**

myCompass users logged in an average of 6 times and completed an average of .29 modules. Healthy Lifestyles users logged in an average of 4 times and completed an average of 1.37 modules. At baseline, mean scores on several outcome measures, including the primary outcome of work and social functioning, were near to the normal range, despite an extensive recruitment process. Approximately 61.6% (473/780) of participants completed the postintervention assessment. Intention-to-treat analyses revealed improvement in functioning, depression, anxiety, diabetes distress, and healthy eating over time in both groups. Except for blood glucose monitoring and medication adherence, there were no specific between-group effects. Follow-up analyses suggested the outcomes did not depend on age, morbidity, or treatment engagement.

**Conclusions:**

Improvement in social and occupational functioning and the secondary outcomes was generally no greater for myCompass users than for users of the control program at 3 months postintervention. These findings should be interpreted in light of near-normal mean baseline scores on several variables, the self-selected study sample, and sample attrition. Further attention to factors influencing uptake and engagement with mental health treatments by people with T2DM, and the impact of illness comorbidity on patient conceptualization and experience of mental health symptoms, is essential to reduce the burden of T2DM.

**Trial Registration:**

Australian New Zealand Clinical Trials Registry ACTRN12615000931572; https://www.anzctr.org.au/Trial/Registration/TrialReview.aspx?id=368109&isReview=true (Archived by WebCite at http://www.webcitation.org/7850eg8pi)

## Introduction

### Background

Type 2 diabetes mellitus (T2DM) is a global public health problem, affecting more than 370 million people worldwide [1,2]. The disease is the fastest growing chronic condition in Australia, with approximately 1 million people diagnosed and up to 500,000 undiagnosed [3]. In addition to increased risk of adverse health consequences (including macro and microvascular diseases), people diagnosed with T2DM are at greater risk of psychiatric and neurodegenerative disorders, as well as physical disability and functional decline, than the general population [4,5]. With the prediction that global prevalence rates will increase to nearly 600 million by 2035 [3], T2DM presents a substantial escalating challenge for health care delivery and public health infrastructure in Australia and around the globe.

Depression is frequently comorbid with T2DM, affecting up to 40% of people with the disease [6,7]. Comorbid depression is associated with greater disease morbidity, mortality, and health care costs. These relationships are likely because of poor occupational and social functioning, reduced regimen adherence, poor quality of life, and increased need for outpatient and inpatient health services in people with both disorders [6,8,9]. The interrelationship between T2DM and depressive symptoms [10] further complicates the situation, with research showing that each condition increases the negative functional and health impacts of the other [6]. A population-based early intervention program for depression in people with T2DM is urgently needed to reduce both the substantial personal burden and public health impact of these comorbid conditions.

International diabetes treatment guidelines now recommend regular screening for depressive symptoms, with referral to appropriate psychological treatments as part of standard diabetes care [11]. Cognitive behavioral therapy (CBT) is the most widely validated treatment for depression. Recent reviews show positive impacts of face-to-face CBT on depressive symptoms, quality of life, fasting glucose levels [12], and self-care [13] in people with T2DM. However, the majority of people with T2DM are cared for in the primary care setting, and depression screening in primary care is highly variable. Within a standard-length consultation, it is difficult for general practitioners to focus on mental health as well as glycemic control and prevention of diabetes complications; in addition, many patients are reluctant to accept a referral for face-to-face psychotherapeutic interventions [14]. Concerns about confidentiality, stigma, treatment cost, and time and lifestyle constraints are further barriers to patients’ help seeking, and psychological services are often scarce in rural and remote areas. In addition, the sizeable base rates of comorbid T2DM and depressive symptoms [15] mean that improved screening and diagnosis will likely place further pressure on face-to-face psychological services, many of which already struggle to meet the needs of their community [16,17]. Therefore, testing more flexible and scalable models of mental health service delivery for people with T2DM is necessary [12].

Internet delivery of evidence-based psychological therapies is now established as apopular, clinically effective, and cost-efficient means of upscaling access to psychological treatments. In people with T2DM, both diabetes-specific [18,19] and generic (ie, not diabetes sensitive but with therapist guidance) [20] depression interventions have yielded positive treatment effects for both depression symptoms and diabetes-related distress. There is also some suggestion that these may provide a potentially cost-effective solution to the substantial disability resulting from depression-diabetes comorbidity [21]. The current literature suggests that such interventions are most effective for people experiencing mild-to-moderate depressive symptoms [22]. As subclinical depression is more prevalent in T2DM than severe depression [23,24], testing of low-intensity Web-based interventions in T2DM patients with mild-to-moderate depressive symptoms is a public health priority.

myCompass is a broadly available Web-based CBT program available free of charge in Australia. A previous trial reported symptom reduction of mild-to-moderate depression in the general population compared with a placebo condition [25]. Despite being self-guided and transdiagnostic (ie, not diabetes specific) in therapeutic content, an uncontrolled feasibility study suggested that myCompass may potentially improve functional and mental health outcomes in people with diabetes [26]. As such, it may provide a more flexible, scalable alternative to disease-specific interventions and may benefit people with T2DM whose depressive symptoms do not warrant more intensive face-to-face intervention and/or who wish to manage their mental health themselves.

### Objectives

The primary aim of this randomized controlled trial (RCT) was to evaluate the effectiveness of myCompass in improving work and social functioning—a major contributor to the T2DM burdens [8]—in adults with T2DM and mild-to-moderate depressive symptoms. On the basis of our feasibility data, we hypothesized that participants using the myCompass intervention would show improved scores on a self-report measure of work and social functioning relative to participants using an active placebo program. The secondary aim was to determine the effectiveness of myCompass for improving a range of diabetes-specific outcomes linked with depression and also shown to impact health outcomes in T2DM, including diabetes-related distress, diabetes self-care, and anxiety symptoms. The inclusion of these variables enabled us to examine whether treatment of depression with a public health intervention was capable of impacting diabetes-specific outcomes.

## Methods

### Design

This study is the primary outcomes evaluation of a 2-arm RCT called *SpringboarD*. The full protocol for the SpringboarD Trial is published elsewhere [27]. In the full SpringboarD trial, outcomes will be assessed at baseline and 3, 6, and 12 months postrandomization. This paper reports data from baseline and 3 months postintervention. Participants in the active and control groups had uninterrupted access to usual treatment for their diabetes throughout the study. This study was approved by Human Research Ethics Committee (HREC) at University of New South Wales (UNSW) Sydney (HREC 15090) and registered with the Australia and New Zealand Clinical Trials Registry (ACTRN12615000931572).

### Participants and Setting

The study utilized a broadly-based recruitment strategy, including offline and online recruitment methods, to enroll the required sample size for sufficient statistical power. Recruitment began in September 2015 and continued until November 2017. Offline recruitment occurred via letters from participating general practices in New South Wales (NSW) and Victoria to their patients with T2DM, distribution of promotional materials in general practice settings throughout NSW and Victoria (eg, study flyers and posters), and print advertisements in national diabetes-related publications.

Online recruitment involved a range of techniques targeting health professionals and individuals. Health professionals were targeted through member associations such as the Australian Association of Practice Managers, the Australian Diabetes Educators Association, the Australian Association of Practice Nurses and the Australian Primary Health Care Nurses Association. Contact was via electronic direct mail, informing members of the study and inviting them to refer appropriate candidates to the SpringboarD website for screening.

Individuals were recruited via Google and Facebook advertisements targeting an Australia-wide audience aged 18 years and over, with interest in *diabetes mellitus type 2 awareness*, *diabetes type 2 awareness* and/or *diabetes awareness*. Advertisements provided a click-through link to a dedicated page on Black Dog Institute’s website from which the SpringboarD website could be accessed for information and screening. Potential candidates were also contacted via email through Black Dog Institute’s Volunteer Research Register (VRR) and the Sax Institute’s *45 and Up Study*. The VRR distributed 2378 emails to research volunteers aged between 18 and 75 years with a history of depressive symptoms. The 45 and Up Study is a large, longitudinal population-based cohort study of healthy aging in NSW, Australia, described in detail elsewhere [28]. The 267,153 study participants are considered largely representative of the Australian population. From June 2017 to September 2017, 4175 participants from the *45 and Up Study* aged between 45 and 75 years with self-reported diabetes were emailed invitations to participate in the SpringboarD study. All promotional material directed potential candidates to a secure study-specific website [29], which guided interested participants through the consent process and provided instructions regarding completion of the screening questionnaires.

### Eligibility Criteria

People were eligible to take part if they were aged 18 to 75 years, diagnosed with T2DM by a physician, screened positive for depression on the self-report 2-item Patient Health Questionnaire (PHQ-2; ie, ≥2) [30], and had access to an internet-enabled device (eg, computer, tablet, and/or mobile phone). People who screened positive for depression completed the 9-item PHQ (PHQ-9) [31] at screening so that the level of symptom severity could be determined. Exclusion criteria included insufficient English literacy, extremely severe depressive symptoms on the full PHQ-9 (score >19), probable psychosis (measured by the psychosis screener developed for the Australian National Mental Health and Wellbeing Survey) [32], currently receiving face-to-face counseling or therapy for depression, change in antidepressant medication within the previous 2 months, high suicide risk (assessed by the PHQ-9 Item-9), and previous use of the myCompass program.

Eligible participants received immediate feedback of their suitability for the study via the website and were provided onscreen instructions for completion of the baseline assessment. Approximately 25 min were required to complete the screening and baseline assessment. All data captured by the study website during the screening, baseline, and postintervention assessments were stored in password-protected files on secure servers that comply with UNSW HREC privacy regulations regarding online data collection.

### Randomization

Computerized block randomization with blocks of 8 was undertaken to assign participants to the intervention and control condition at a 1:1 allocation ratio. Randomization was initiated immediately after the completion of the baseline assessment by the automated randomization system built into Black Dog Institute’s study management software. Allocation was concealed from participants and researchers. Though participants were ineligible if they had previously used myCompass, some participants allocated to the intervention condition may have independently accessed public information about myCompass over the course of the trial and therefore become aware that they were allocated to an existing Web-based therapy program.

### Interventions

#### Active Intervention (myCompass)

**Figure 1:**
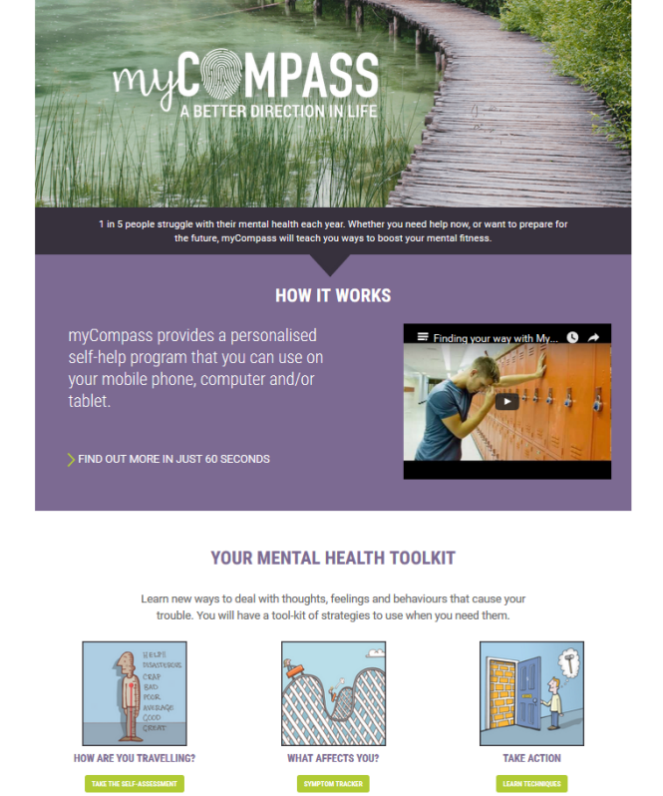
Screenshot of the myCompass landing page.

**Figure 2:**
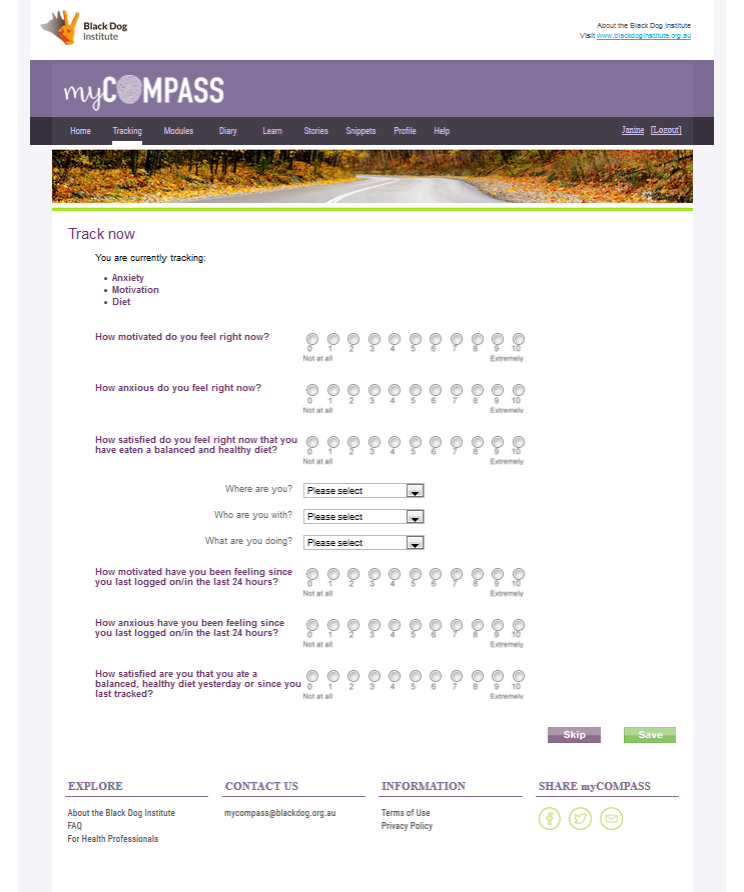
Screenshot of the myCompass self-monitoring page.

myCompass (mycompass.org.au) is a public health fully automated, self-guided CBT intervention that users complete in their own time and at their own pace on their computer and/or mobile phone (see Figures 1 and 2).

The program offers a personalized treatment plan based on an assessment of user symptoms, either at registration or at any time during program use [25,27]. The core program consists of 12 interactive CBT modules and 20 cognitive and/or behavioral variables for self-monitoring. For maximum outcomes, users are recommended to complete 3 modules and 3 self-monitoring variables. The myCompass program offers flexibility for users to select their own CBT modules and self-monitoring variables, or, if they prefer, algorithm-based guidance. It provides access to a range of other resources including short message service (SMS) text messaging and/or email self-monitoring reminders, home practice activities to facilitate skill generalization, mental health care tips and motivational statements delivered by email/SMS text messaging, and graphical reporting of self-monitoring data. Registering to use myCompass is free, and users are not billed for the SMSs they receive.

Participants randomized to the myCompass arm were provided access to the full program for 8 weeks. The program recommended that users complete 3 CBT modules and self-monitor up to 3 symptoms or behaviors. A 4-week tailing-off period followed, in which only the symptom monitoring function was accessible. Studies have shown that the use of Web-based interventions is improved when users receive program feedback that is personalized in its content [33]. For this reason, myCompass users receive automated and personalized feedback via email about their use of the program’s self-monitoring and module functions in weeks 1, 3, 5, and 7.

myCompass user privacy is managed by a password-protected log-on and by ensuring that user-generated data (eg, self-monitoring ratings) are not stored on the user’s device but are instead transferred via the internet using secure sockets layer protocols (which encrypt transmitted data by rendering data unreadable to anyone other than the intended recipient) and by storing the data in secure servers. Participant user data were identified in myCompass using email details provided during study registration. Once extracted, data were deidentified and stored in a password-protected file.

#### Placebo Intervention (Healthy Lifestyles)

The placebo control program, *Healthy Lifestyles*, was adapted from a control program used in previous studies by members of the research team [34] to replicate the mode of delivery and key functionality of myCompass, but without the therapeutic content. The program contains 12 modules that deliver health and lifestyle information across a range of topics (eg, skin care and mobile phone hygiene), interactive exercises, and the potential for program tailoring via a brief survey completed at registration. Program users received an email at weeks 1, 3, 5, and 7, containing a brief reminder to log into the program but no feedback about their program use. They also received a weekly SMS text message containing a fact relevant to the content of Healthy Lifestyles for the first 4 weeks of the intervention period to match the SMS functionality of myCompass. The Healthy Lifestyles program was designed to have high face validity as a health and lifestyle intervention without any symptomatic benefit [34]. Participants had full access to the Healthy Lifestyles program for 8 weeks. The core features of each program are presented in Table 1.

### Outcome Measures

A summary of the measures obtained from participants at baseline and 3 months postintervention is presented in Table 2.

**Table 1:** A comparison of the core features of the myCompass and Healthy Lifestyles programs.

Feature	MyCompass	Healthy Lifestyles
Modality	Website	Website
Symptom tracking	Yes: track up to 3 symptoms	Not available
Usage reminders	Yes: automated short message service text message or email	Yes: automated email
Number of modules	12: including interactive learning activities	12: including interactive learning activities
Module content	Cognitive and behavioral strategies for mood, anxiety, stress, and mental well-being	Generic health literacy information

**Table 2:** Measures obtained at each assessment phase.

Measure		Time 1		Time 2	
**Demographic and disease-related information**					
	Demographic data (eg, age and gender)		X		—^a^
	Disease-relevant data (eg, age at diagnosis and treatment)		X		X
	Mental health history (eg, previous diagnoses and help-seeking)		X		—
**Primary outcome**					
	Work and Social Adjustment Scale [31]		X		X
**Secondary outcome**					
	Patient Health Questionnaire-9 [26]		X		X
	Diabetes Distress Scale [33]		X		X
	Generalized Anxiety Disorder Scale [34]		X		X
	Self-management Profile for Type 2 Diabetes Scale (behavior items only) [35]		X		X
	Glycosylated hemoglobin		X		—
	Days out of role [36]		X		X
	Health service utilization		X		X

^a^Not applicable.

#### Primary Outcome

The primary outcome for the trial was a between-group difference in work and social functioning, measured by the Work and Social Adjustment Scale (WSAS). The WSAS is a psychometrically sound measure of daily functioning across 5 domains, including work, social leisure activities, private leisure activities, home management, and personal relationships [35,36]. Scores range from 0 to 40, with higher scores indicating poorer functioning.

#### Secondary Outcomes

Symptoms of depression and anxiety were measured by PHQ-9 [31] and the 7-item Generalized Anxiety Disorder scale (GAD-7) [37], respectively. Both scales are well validated, are used widely as screening tools in primary care settings and are frequently included as outcome measures in studies of Web-based interventions [38,39]. Both scales use cut-off scores of 5, 10, and 15 to identify people with mild, moderate, and moderately severe symptoms, respectively.

Emotional adjustment to diabetes—or *diabetes-related distress* —was measured by the 17-item Diabetes Distress Scale (DDS) [40]. The DDS yields a total score plus 4 subscale scores assessing the perceived emotional burden of diabetes, along with physician-related distress, regimen-related distress, and interpersonal distress. DDS total and subscale scores are calculated as the average across all items in the scale/subscale and range from 1 to 6, with higher scores indicating greater distress. A score of >3 indicates clinically relevant distress.

Diabetes self-care was assessed using a subset of items from the Self-Management Profile for Type 2 Diabetes (SMP-T2D). The SMP-T2D was designed for use in clinical trials to assess level and perceived ease of performance of specific T2DM regimen behaviors. Perceived coping and confidence dealing with diabetes and ease of weight management are also assessed, and all constructs demonstrate appropriate internal consistency, validity, and sensitivity [41]. To avoid redundancy (eg, the DDS also asks about difficulties with self-care activities), to reduce participant assessment burden, and as we were primarily interested in learning about participants’ behavioral engagement in self-care activities, we only administered items that measured the level of self-care across 4 patient behavior domains: blood glucose monitoring, medication adherence, healthy eating, and physical activity. Scores in each behavior domain are converted to a percentage of the previous week spent engaging in a particular self-care behavior. Higher scores indicate greater time spent on self-care [41].

### Additional Measurements

At baseline, we collected disease-related (eg, age of onset and treatment regimen), sociodemographic (eg, age, gender, education, and occupation) and mental health history data (eg, service use and previous diagnoses). We also obtained participants’ most recent glycosylated hemoglobin (HbA_1c_) from their medical records as an indicator of their overall blood glucose management before study commencement. Recent service utilization for physical and mental health problems was assessed at baseline and postintervention, along with days out of role, defined as the number of days in the previous 30 days that a participant was unable to perform work or normal activities because of problems with his/her physical or mental health [42].

At the conclusion of the intervention period, program engagement data were extracted for myCompass and Healthy Lifestyles, including frequency of log-in, number of modules started and completed, and self-monitoring frequency (myCompass only).

### Sample Size

Power calculations indicated that a study sample of 600 was needed to detect a minimum difference of .3 standard deviations between groups in mean change in scores on the WSAS at 3 months post intervention, with power of 80%, 2-tailed alpha=.05, and assuming an attrition rate of 40%. Owing to early indications of a higher attrition rate at postintervention than anticipated, a further 180 people were recruited into the study to ensure sufficient power to test the research hypotheses.

### Analyses

Primary analyses employed an intention-to-treat (ITT) approach using mixed-model repeated measures analyses (MMRM) computed within the Mixed procedure of SPSS version 23 (IBM Corp.). MMRM makes use of all available data to obtain parameter estimates and is widely recognized as an appropriate strategy for analyzing incomplete datasets [43]. In this study, restricted maximum likelihood estimation was employed to estimate model parameters, and error degrees of freedom were calculated using Satterthwaite approximation. In line with Fairclough and Helms’ [44] recommendation that the covariance structure be restricted in situations of high attrition, analyses assumed a compound symmetric structure. Repeated measures (Level 1) were nested within individuals (Level 2), and a random intercept was used at the individual level to account for intraindividual correlations on repeated measures.

In addition to the ITT analyses, we conducted completer analyses to examine the effects of treatment on those individuals who completed the study, defined as having provided complete data at 3-month follow-up. In these analyses, individuals with any missing data at 3-month follow-up were deleted case wise (myCompass n=175; Healthy Lifestyles n=151), and repeated-measures analyses of variance were conducted for each of the primary and secondary outcomes on the remaining participants. As the sample characteristics and treatment effects in the completer analyses did not differ from those of the ITT analyses, only the ITT results are reported.

## Results

### Sample Characteristics

**Figure 3:**
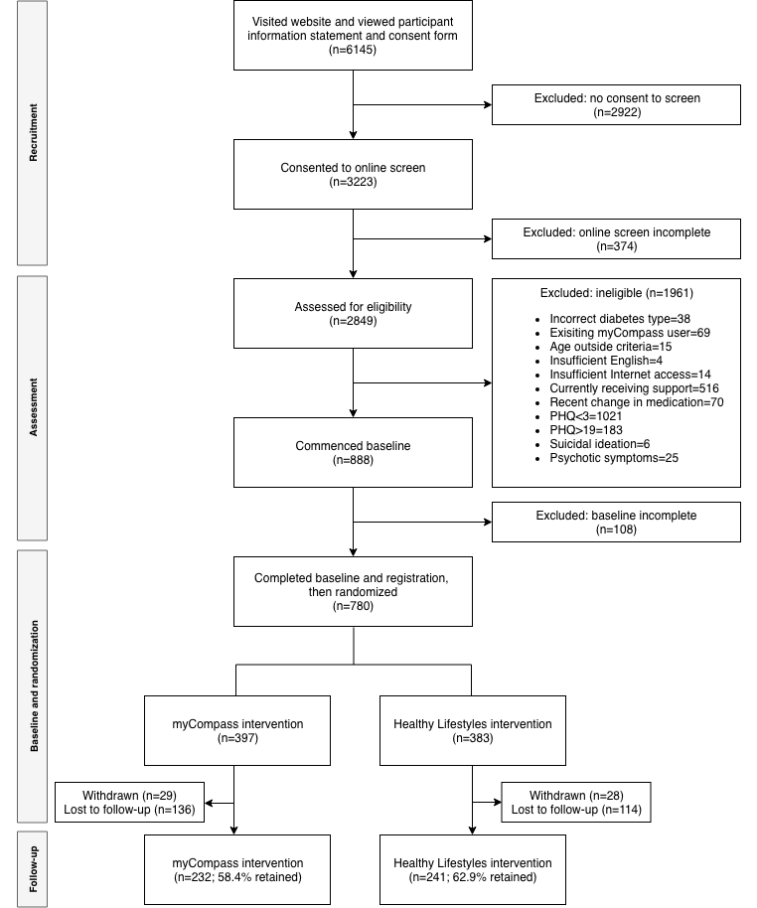
Consolidated Standards for Reporting of Trials diagram of participant flow through the SpringboarD trial. PHQ: Patient Health Questionnaire.

Overall sample characteristics are presented in Table 3, and participant flow through the study is presented in Figure 3. Of the 6145 visits to the SpringboarD Project website, 3223 consented to Web-based screening, yielding 888 eligible participants who commenced the baseline assessment. The main reasons for ineligibility included the following: did not meet inclusion criteria for the presence of depressive symptoms, that is, a score of <2 on the PHQ-2 (52.07% [1021/1961]), currently receiving face-to-face mental health support (26.31% [516/1961]), and screening results indicating severe depression (9.33% [183/1961]). A total of 780 individuals completed the baseline assessment and were randomized. A total of 57 people subsequently withdrew study consent, leaving a final study sample of 723 individuals.

As shown in Table 3, groups were well matched between the trial arms. The myCompass group reported stable antidepressant use of somewhat longer duration than the Healthy Lifestyles group, with no other notable differences in demographics and mental health. The overall sample was predominantly female (68.8% [498/723]), married (55.7% [408/723]), employed at least part time (50.8% [367/723]), university educated (34.02% [246/723]), with an average age of 58 years (SD 10.35). More than 3 quarters of the sample (83.9% [607/723]) had at some time sought professional help for common mental health issues (eg, low mood, anxiety and/or stress), and almost half (43.8% [317/723]) had previously received at least one mental health diagnosis, the most frequent being depression (40.9% [296/723]).

**Table 3:** Sample characteristics for myCompass and Healthy Lifestyles groups.

Characteristic			myCompass (N=368)		Healthy Lifestyles (N=355)	
**Demographics**						
	Age, mean (SD)		57.7 (10.6)		57.7 (10.0)	
	Female (n=465), n (%)		229 (62)		236 (66)	
	Married (n=387), n (%)		204 (55)		183 (52)	
	Employed (n=351), n (%)		173 (47)		178 (50)	
	**Education level, n (%)**					
		Secondary school or lower (n=220)		112 (30)		108 (30)
		Trade certificate or diploma (n=270)		133 (36)		137 (39)
		University undergraduate or more (n=233)		123 (33)		110 (31)
**Mental health**						
	**Lifetime history, n (%)**					
		Sought professional support for mental health (n=571)		296 (80)		275 (77)
		Received mental health diagnosis (n=300)		155 (42)		145 (41)
		Diagnosed with depressive symptoms or major depressive disorder (n=279)		143 (38)		136 (38)
	**Past 6 weeks, n (%)**					
		Sought professional support for mental health (n=113)		65 (18)		48 (14)
	**Current, n (%)**					
		Taking antidepressant medication (n=241)		125 (34)		116 (33)
		Months using antidepressant medication		97.70 (94.72)		73.67 (56.21)^a^
**Diabetes**						
	Age at diagnosis, mean (SD)		46.6 (11.1)		47.2 (10.9)	
	**Diabetes treatment, n (%)**					
		Healthy eating (n=437)		230 (63)		207 (58)^a^
		Physical activity (n=323)		176 (48)		147 (41)
		Oral medication (n=583)		295 (80)		288 (81)
		Insulin (n=216)		113 (31)		103 (29)
		Exenatide (n=32)		21 (6)		11 (3)
	**Past 6 weeks, n (%)**					
		Visited general practitioner for diabetes (n=419)		218 (59)		201 (57)
		Frequency of general practitioner visit		1.31 (.78)		1.37 (.71)
		Hospitalized for diabetes (n=24)		13 (4)		11 (3)
		Frequency of hospitalization for diabetes		1.46 (1.5)		1.36 (.9)

^a^Means differ significantly at *P*<.05.

Diabetes-related characteristics were also largely similar between the groups. The average age of onset of T2DM for the sample was 47 years (SD 10.84), and for the 60.0% (434/723) of participants for whom HbA_1c_ data were available from medical records, the mean value was 7.5% (SD 1.6).

Baseline scores for key outcome measures for both groups are presented in Table 4. Again, randomization was largely successful, with 2 exceptions noted for the WSAS (*F*_1, 782_=5.807; *P*=.02; *d*=0.17) and SMP-T2D Healthy Eating Domain (*F*_1, 782_=15.925; *P*=.03; *d*=0.29). However, when the relevant normative cut-offs were applied, baseline scores on the key outcome variables were near to the normal range. WSAS scores were at the lower end of the *significant impairment* range of 10 to 20 [35] (sample mean 12.89), PHQ-9 scores were slightly above the most common diagnostic cut-off of 10 [31] (sample mean 11.06), scores on the DDS were below the recommended clinical cut-off of ≥3 [40] (sample mean 2.54), and scores on the GAD-7 were also below the recommended clinical cut-off of ≥3 [37] (sample mean 7.45).

### Intention-to-Treat Analyses (Mixed-Model Repeated Measures Analyses)

The observed scores for all study outcomes postintervention are presented in Table 4, and the group and time fixed effects from the MMRM analyses are shown in Table 5. Irrespective of the intervention received, all participants reported a significant reduction in WSAS scores across time. Similarly, all participants showed symptomatic improvements on the PHQ-9, DDS, and GAD-7 between baseline and postintervention, with no between-group differences detected posttreatment on any of these measures. The average mean reductions across time were generally small and did not meet any recommended threshold for clinical significance.

For the SMP-T2D, no group or time differences were observed for the Physical Activity Domain, and both groups reported significant improvement in the Healthy Eating Domain. Scores on the Blood Glucose Monitoring Domain and Medication Adherence Domain increased over time for the Healthy Lifestyles group relative to the myCompass group, with Healthy Lifestyles participants increasing time spent each week monitoring blood glucose and taking medication as recommended by approximately .4 to .5 days, compared with their myCompass counterparts.

### Posthoc Treatment Effect Moderation Analyses

Level of glycemic control [45] and severity of depression symptoms [46] are potential moderators of treatment effects, and age remained a consistent predictor of overall symptomatic improvement in our sample. We therefore examined the possible moderating role of these variables by repeating our MMRM approach with an additional 3-way interaction term that included the moderator variable of interest (ie, Group×Time×Moderator). Depressive symptoms were dichotomized into “clinical” and “subclinical” groups on the basis of the PHQ-9 diagnostic cut-off of 12 recommended for individuals with diabetes [31]. Level of glycemic control was determined using conventional HbA_1c_ targets for T2DM, with >7% indicative of suboptimal control and ≤7% indicative of well-controlled diabetes [47]. Age was grouped using a median split. No moderator demonstrated a significant impact on treatment effects (all *P*>.05).

**Table 4:** Baseline and postintervention means (SDs) on key outcome variables for myCompass and Healthy Lifestyles groups.

Measure	Baseline, mean (SD)		Post (3 months), mean (SD)	
	myCompass (n=397)	Healthy Lifestyles (n=387)	myCompass (n=232)	Healthy Lifestyles (n=241)
WSAS^a^	13.64^b^ (7.93)	12.23^b^ (7.77)	12.24 (9.31)	10.82 (9.19)
PHQ-9^c^	11.30 (4.03)	10.86 (4.11)	8.68 (5.63)	8.24 (5.54)
GAD-7^d^	7.60 (4.10)	7.33 (4.20)	6.66 (4.73)	6.20 (4.58)
DDS^e^	2.56 (0.97)	2.56 (0.95)	2.24 (0.99)	2.18 (0.95)
SMP-BG^f^	49.61 (39.50)	45.55 (38.85)	49.25 (39.33)	55.83 (40.21)
SMP-MA^g^	87.50 (22.47)	87.20 (22.82)	86.34 (24.15)	92.04 (15.12)
SMP-HE^h^	49.96^b^ (29.16)	48.26^b^ (28.61)	53.52 (28.94)	54.87 (28.25)
SMP-PA^i^	53.44 (33.48)	48.26 (33.61)	55.98 (34.64)	52.44 (34.07)

^b^Means differ significantly at *P*<.05.

^a^WSAS: Work and Social Adjustment Scale.

^c^PHQ-9: Patient Health Questionnaire.

^d^GAD-7: Generalized Anxiety Disorder Scale.

^e^DDS: Diabetes Distress Scale.

^f^SMP-BG: Self-Management Profile for Type 2 Diabetes—Blood Glucose Monitoring.

^g^SMP-MA: Self-Management Profile for Type 2 Diabetes—Medication Adherence.

^h^SMP-HE: Self-Management Profile for Type 2 Diabetes—Healthy Eating.

^i^SMP-PA: Self-Management Profile for Type 2 Diabetes—Physical Activity.

**Table 5:** Mixed-model repeated measures analyses fixed effects for time, group, and time×group on key outcome variables.

Variable and effect		Beta^a^		SE		*t* test (*df*)		*P* value		95% CI	
**WSAS^b^**											
	Time		1.344		0.48		2.800 (544.48)		.005^c^		0.401 to 2.287
	Group×Time		0.196		0.684		0.287 (550.76)		.77		–1.147 to 1.540
**PHQ-9^d^**											
	Time		2.395		0.31		7.736 (596.32)		<.001^c^		1.787 to 3.003
	Group×Time		0.144		0.44		0.326 (604.61)		.74		–0.720 to 1.008
**DDS^e^**											
	Time		0.302		0.047		6.361 (518.94)		<.001^c^		0.208 to 0.395
	Group×Time		–0.062		0.068		–0.919 (523.64)		.36		–0.194 to 0.070
**GAD-7^f^**											
	Time		1.009		0.264		3.825 (553.83)		<.001^c^		0.490 to 1.526
	Group×Time		–0.276		0.375		–0.736 (560.75)		.46		–1.013 to 0.460
**SMP-BG^g^**											
	Time		–8.253		2.278		–3.623 (538.46)		<.001^c^		–12.727 to –3.778
	Group×Time		8.98		3.248		2.764 (545.46)		.006^c^		2.598 to 15.360
**SMP-MA^h^**											
	Time		–3.126		1.24		–2.520 (490.84)		.01^c^		–5.562 to –0.688
	Group×Time		5.117		1.763		2.901 (496.65)		.004^c^		1.651 to 8.582
**SMP-HE^i^**											
	Time		–5.704		1.604		–3.553 (527.04)		<.001^c^		–8.856 to –2.550
	Group×Time		3.266		2.282		1.430 (532.61)		.15		–1.217 to 7.750
**SMP-PA^j^**											
	Time		–3.440		2.228		–1.543 (559)		.12		–7.816 to 0.936
	Group×Time		0.083		3.165		0.541 (566.75)		.98		–6.301 to 6.134

^a^Beta: unstandardized regression coefficient for the effect holding constant age and sex.

^b^WSAS: Work and Social Adjustment Scale.

^c^Significant at *P*<.05.

^d^PHQ-9: Patient Health Questionnaire-9 item.

^e^DDS: Diabetes Distress Scale.

^f^GAD-7: Generalized Anxiety Disorder Scale-7 item.

^g^SMP-BG: Self-Management Profile for Type 2 Diabetes—Blood Glucose Monitoring.

^h^SMP-MA: Self-Management Profile for Type 2 Diabetes—Medication Adherence.

^i^SMP-HE: Self-Management Profile for Type 2 Diabetes—Healthy Eating.

^j^SMP-PA: Self-Management Profile for Type 2 Diabetes—Physical Activity.

### Study Attrition

Of the total trial participants, 59.2% (235/397) of the participants from the myCompass program and 63.3% (245/387) of the participants from the Healthy Lifestyles program provided at least one postintervention measure. A multivariate analysis of variance comparing nonresponders (ie, participants who did not provide any postintervention measures) with responders at baseline revealed differences on several key outcome variables. Nonresponders reported more severe depressive symptoms (*F*=8.362; *P*=.004; *d*=0.27), more severe anxiety symptoms (*F*=3.845; *P*=.05; *d*=0.18), greater diabetes-related distress (*F*=9.095; *P*=.003; *d*=0.28) and poorer medication adherence (*F*=6.564; *P*=.011; *d*=0.19). In a follow-up logistic regression predicting responder status from baseline WSAS, PHQ-9, GAD-7, DDS, and SMP-T2D scores, PHQ-9 (beta=–.058; *P*=.021; OR [odds ratio] .943; 95% CI [.898, .991]), DDS (beta=–.221; *P*=.026; OR 1.029; 95% CI [.660, .974]) and WSAS (beta=.029; *P*=.016; OR .802; 95% CI [1.005, 1.054]) scores independently predicted nonresponse. Though effects were generally small, it appears that levels of distress and functioning had some influence on participants remaining in the study.

### Program Use and Feedback

Participants in the treatment group logged in to myCompass an average of 6 times (SD 9.01; range 1-71), started a mean of 0.71 modules (SD 1.18; range 0 to 8), fully completed an average of .29 modules (SD .87; range 0-7), and monitored their symptoms an average of 2 times (SD 5.79; range 0-53). Participants in the control group logged in to Healthy Lifestyles an average of 4 times (SD 3.22; range 1 to 17), started a mean of 2.61 (SD 2.78; range 0 to 8), and completed an average of 1.37 modules (SD 2.24; range 0 to 8). Participants who logged in to their assigned Web-based program did not differ significantly from those who did not, except for a slightly higher anxiety score on the GAD-7 among myCompass users who logged in (*F*=10.76; *P*=.001; *d*=0.39). Adherence indices did not correlate with baseline scores on any primary or secondary outcome. Approximately 54.7% (127/232) of myCompass participants and 11.2% (27/241) of Healthy Lifestyles participants reported that, overall, they found their assigned Web-based program both easy and convenient to use.

## Discussion

### Principal Findings

This trial examined the effectiveness of a self-help, unguided Web-based CBT program (myCompass) for improving work and social functioning in people with T2DM and mild-to-moderate depressive symptoms compared with an active placebo intervention. Our ITT analyses showed that participants’ work and social functioning improved significantly postintervention, irrespective of the intervention received. Significant improvements were also observed for both groups in depressive and anxiety symptoms, diabetes distress, and most aspects of diabetes self-management. Our Healthy Lifestyles group showed small but significant improvements in blood glucose monitoring and medication adherence over and above those observed for the myCompass group. As morbidity may influence the outcomes of depression treatments [46] and as age remained a significant outcome predictor in our models, we examined the potential moderator effects on treatment outcomes of age, depressive symptoms, and diabetes control. Analyses revealed that treatment effect estimates were not impacted by these variables.

The absence of differential treatment effects for work and social functioning and depressive symptoms for people with T2DM following treatment with myCompass was surprising in light of previous findings of accelerated symptom gains in myCompass users in the general community [27] and in our pilot study [26]. Although our findings also contrast with previous studies of Web-based depression interventions in people with diabetes [18,20], there are important points of differentiation between this study and previous diabetes trials that need to be considered in interpreting our findings.

Our recruitment methods targeted people with T2DM and mild-to-moderate depressive symptoms to investigate a public health rather than a clinical application of Web-based CBT; however, our final sample unexpectedly comprised a group with minimal symptoms of depression (47% of participants in the *minimal-to-mild* range on the PHQ-9). This contrasts with previous studies of people with diabetes that included only patients with more severe depressive symptoms [18] or those meeting criteria for major depressive disorder (Newby et al, 2017). Moreover, although 58% of our participants reported *impaired* work and social functioning at baseline, scores on the WSAS suggested that the level of impairment experienced by our participant group was again at the milder end of the disability spectrum. In other words, by all measures, our sample was only minimally impaired at baseline.

Even in the absence of true depression, people with chronic illness typically report poorer daily functioning than those without [48]. Consequently, our statistical analyses were based on a participant group with baseline scores on the WSAS and PHQ-9 that were potentially at *floor* for this cohort, and therefore, any improvement was likely to be minor. Also contrasting with previous studies [20], we observed a systematic pattern of attrition, such that increased severity of depressive symptoms was linked to study dropout. Systematic attrition of those in greatest need of intervention, and for whom treatment benefits were likely to be largest, may have further magnified any floor effects in our study [49] and precluded us from finding larger and more significant functional and symptom improvements following treatment with myCompass.

It is important to understand why and how increasing severity of depressive symptoms compromises study involvement in trials of Web-based CBT to inform the take-up of suitable interventions for this patient cohort and to maximize treatment effectiveness. One possibility is that attrition is related to increasing levels of amotivation, concentration difficulties, and behavioral inactivation that are hallmark symptoms of depressive disorders [50]. Alternatively, anhedonia (ie, reduced capacity to experience and anticipate enjoyment from activities) has been shown to compromise reward-seeking behavior and decision making [51] and might interfere with program uptake by rendering user behavior less responsive to reinforcement history (eg, motivational feedback) and anticipatory benefits (eg, information about program effectiveness) [52]. An accumulating body of evidence suggests that analysis of individual or *clusters* of depression symptoms may be necessary for understanding behavioral health outcomes in diabetes patients [53,54]. More precise understanding of relationships between depressive symptoms and indicators of program uptake and treatment benefit in T2DM is a challenge for future research.

Importantly, the myCompass program is a completely self-guided mental health intervention that is generic in content. It is, therefore, lower in treatment intensity than previously studied therapist-guided programs [20], and it lacks the disease specificity of diabetes-themed programs [18,19]. Fisher and colleagues [24] have previously suggested that scores on depression screeners may be less reflective of mood disturbance than general emotional and diabetes-specific distress, that is, distress experienced in response to the daily challenges and demands of living with diabetes. In line with this, we have recently published data showing that mildly depressed adults with diabetes maintain a very nuanced conceptualization of their mental health symptoms in which low mood, anxiety, and stress are generally perceived as features of the diabetes diagnosis (and warranted by contextual stressors) and not separate or comorbid conditions to be managed [55]. Lack of differentiation of mild-to-moderate depression symptoms from adjustment to diabetes has potential to attenuate the personal relevance and clinical effectiveness of interventions targeting broad CBT skills and techniques. At the same time, mild-to-moderate depression covers different levels of symptom severity that may differ in terms of responsiveness to internet-based CBT. Further exploration of the conceptualization of depression in T2DM and associated implications for depression treatment is warranted. Moreover, we recommend that researchers examine the nature of comorbidity between depression and diabetes-related distress. If discrete patterns of depressive symptoms and diabetes-related distress can be distinguished, then different interventions (eg, therapist guided versus unguided, generic versus diabetes specific) may be needed to maximize symptom improvement and increase social and occupational functioning in each.

Of the set of secondary outcomes, the only consistent group effect was for medication adherence, with a small but significant positive effect for the attention control. This finding was isolated and seemingly counterintuitive. However, it is possible that improvement in medication taking was prompted by the focus paid by the attention control program to healthy lifestyle behaviors.

### Strengths and Limitations

Recruitment and retention in RCTs targeting comorbid physical and mental illness can be difficult [56]. Despite a lengthy and comprehensive community recruitment strategy, and our adoption of retention strategies that have been used successfully in other studies, participant enrollment and retention for our trial was challenging (and will be discussed in a future publication). Although we were successful in retaining a sample that afforded us sufficient power to test our research hypotheses, attrition in our study was systematic. As a result, near-normal scores on baseline variables may have influenced program engagement (that was generally low) and weakened tests of treatment effects. Moreover, our data are mostly relevant to people with T2DM experiencing mild functional impairment and mild depressive symptoms. Future studies may benefit from broadening eligibility symptom thresholds. Moreover, research designs that include greater program guidance and feedback (eg, module recommendations and homework follow-up) and provide more regular research support (eg, reminders and encouragement) may be more acceptable to participants with higher levels of impairment, for whom motivational factors are likely to impact ongoing study involvement. Importantly, recruitment and retention in future trials may benefit from further investigation of factors influencing individuals’ decisions to decline trial participation or drop out post study commencement.

Although previous trials of Web-based CBT in people with diabetes have compared the active treatment with either treatment as usual [20] or waitlist control [18], a key strength of our design was the inclusion of an attention-placebo condition using a health literacy tool that had been validated elsewhere [25,34]. However, it is possible that lifestyle information had unexpected relevance to our chronically ill cohort, who were mostly experiencing only minor psychological distress. This may have resulted in increased engagement with the Healthy Lifestyles program and afforded benefit to our control condition in addition to the intended placebo effect. Further assessment with a waitlist and/or treatment-as-usual control group is required.

### Conclusions

In conclusion, this study sought to determine if a broadly available public health Web-based CBT intervention could help individuals with T2DM and mild-to-moderate depressive symptoms improve their daily lives. Functioning and symptom outcomes improved between baseline and postintervention; however, no treatment advantage was observed for the myCompass group. Further research is necessary to identify the factors that impact participant retention in studies of Web-based interventions in T2DM, especially among those in greatest need of psychological support and who stand to benefit most from treatment. At the same time, there is a need to better understand how individuals with diabetes conceptualize mood symptoms in the context of diabetes to ensure that Web-based interventions are both personally and clinically relevant. The personal and societal health burdens posed by comorbid T2DM and depressive symptoms are considerable and will continue to grow. Continued investigation of the potential for Web-based CBT to provide a solution in T2DM is essential.

## Abbreviations

CBT: cognitive behavioral therapy
DDS: Diabetes Distress Scale
GAD: Generalized Anxiety Disorder scale
HbA_1c_: glycosylated hemoglobin
HREC: Human Research Ethics Committee
ITT: intention-to-treat
MMRM: mixed-model repeated measures analyses
NSW: New South Wales
OR: odds ratio
PHQ: Patient Health Questionnaire
RCT: randomized controlled trial
SMP-T2D: Self-Management Profile for Type 2 Diabetes
SMS: short message service
T2DM: type 2 diabetes mellitus
UNSW: University of New South Wales Sydney
VRR: Volunteer Research Register
WSAS: Work and Social Adjustment Scale

## Appendix

Multimedia Appendix 1 CONSORT‐EHEALTH checklist (V 1.6.1).
